# Supplementary figures and images for: Recapitulating human ovarian aging using random walks
Source: PeerJ. 2022 Aug 22;10:e13941. doi: 10.7717/peerj.13941 (PMC9406804; doi:10.7717/peerj.13941)

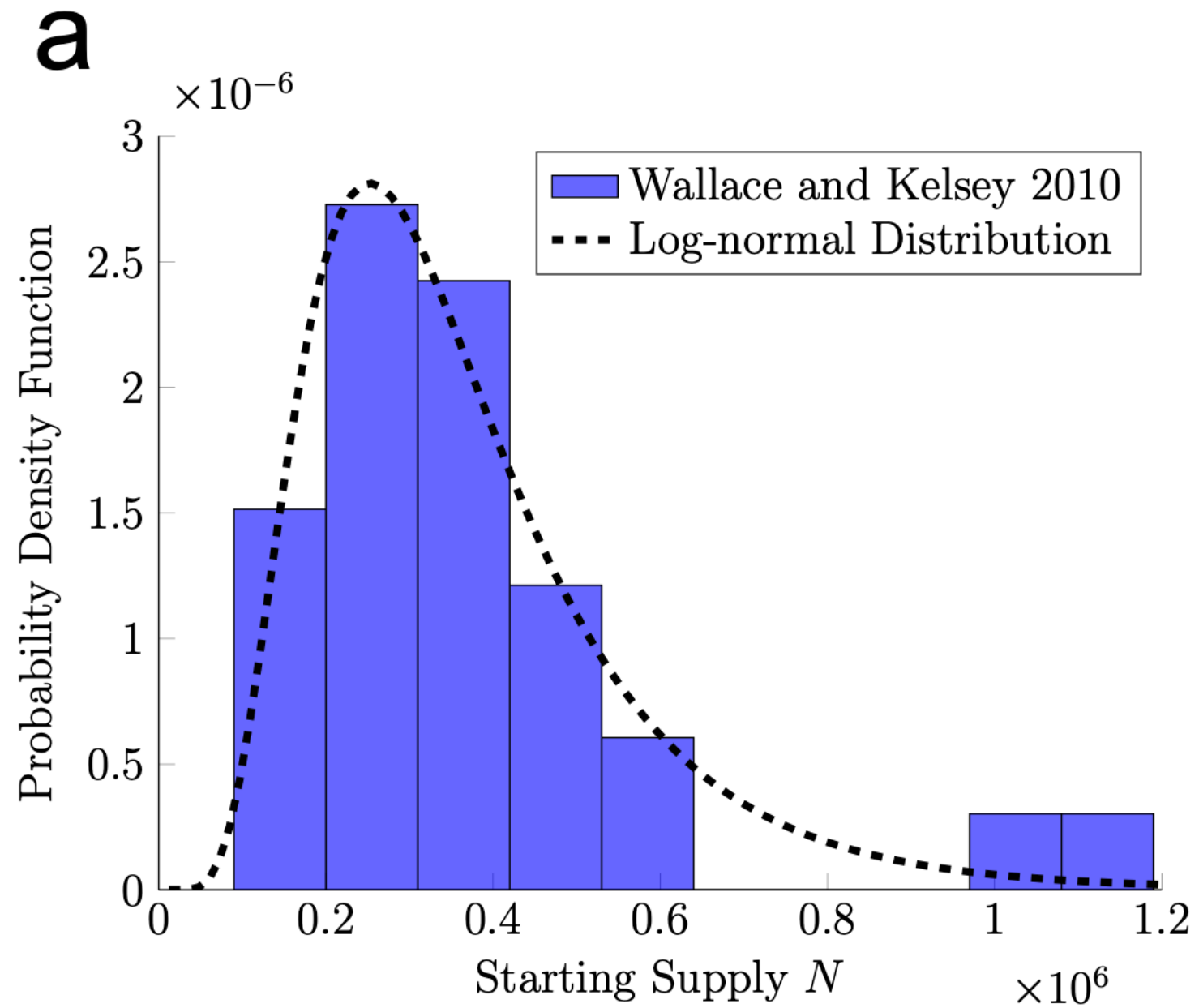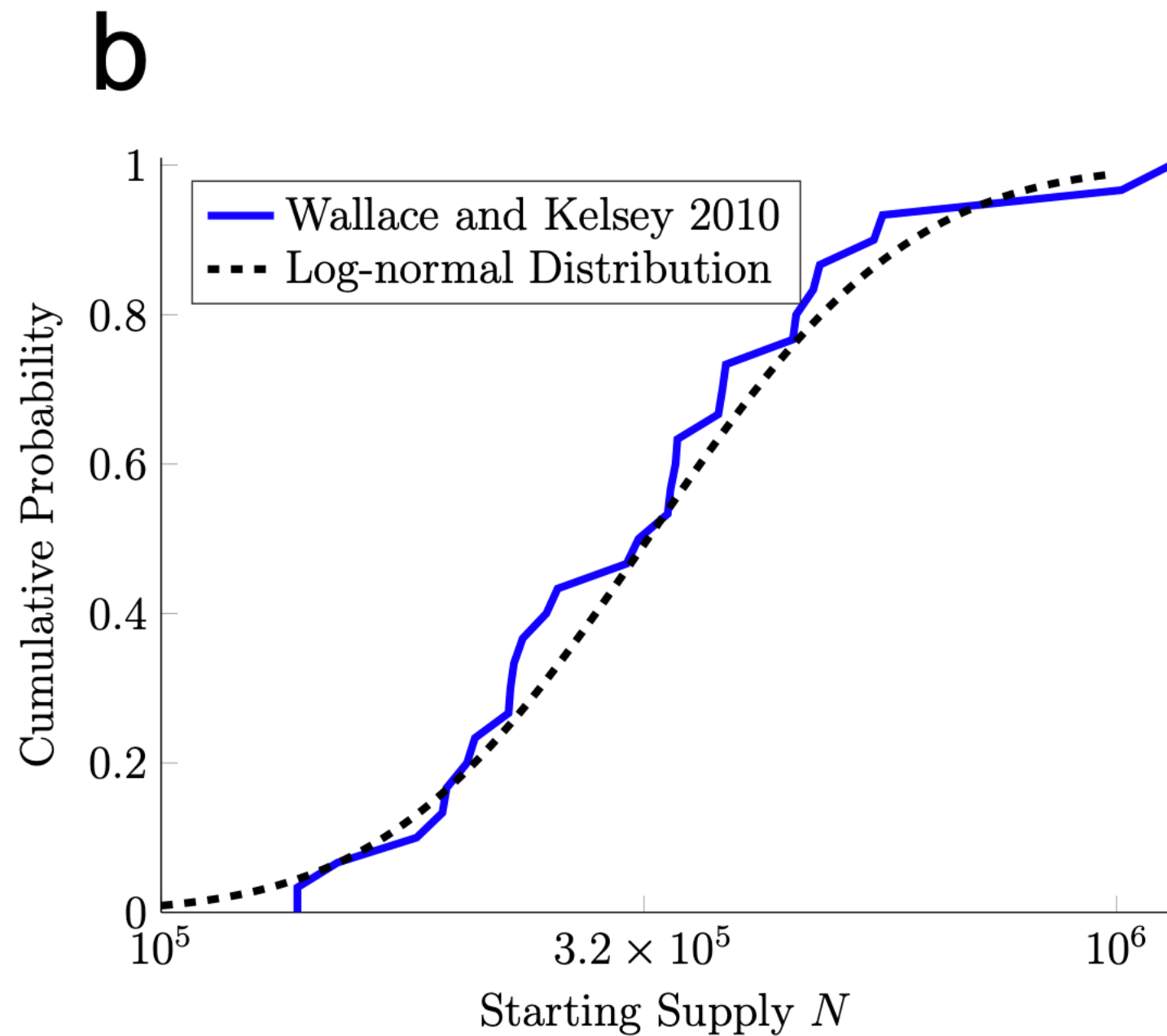

Supplement: Supplemental Information 1 — A) A histogram of the 30 PF counts for women near birth reported by Wallace & Kelsey (2010) (blue bars), which is well-approximated by the log-normal distribution in Eq. (8)–Eq. (9) (dashed black curve). B) A cumulative distribution function plot of observed PF counts (blue solid line) vs. the log-normal distribution (dashed black curve). [file peerj-10-13941-s001.pdf]
