# Supplementary material for: Recapitulating human ovarian aging using random walks
Source: PeerJ. 2022 Aug 22;10:e13941. doi: 10.7717/peerj.13941 (PMC9406804; doi:10.7717/peerj.13941)
Supplement: Supplemental Information 2 — As shown, an ANM distribution centered around a median age of approximately 51 (red vertical line) can be produced in each case, with few simulated subjects reaching menopause before 40 years and after 60 years. Time-Variant drift indicated by the asterisk (*) was applied by modifying drift conditions and also applying a single step drift acceleration in year 38 of simulation time. This was used to interrogate the possibility that PF loss accelerates during reproductive aging. Note that here, the ANM distribution generated when Subject-Variable drift is applied (middle panel) is broader than that seen for homogenous drift (top panel) given otherwise identical model conditions. Application of Time-Variant drift resulted again in a narrower ANM distribution, and prevented simulated subjects from reaching the ANM threshold after age 62 (blue vertical line). [file peerj-10-13941-s002.pdf]

<table>
<tr><th>Drift Parameters (Corresponding Figure)</th><th>ANM Histogram</th></tr>
<tr><td>Subject-Identical, Time-Invariant
(Figure 2a)</td><td>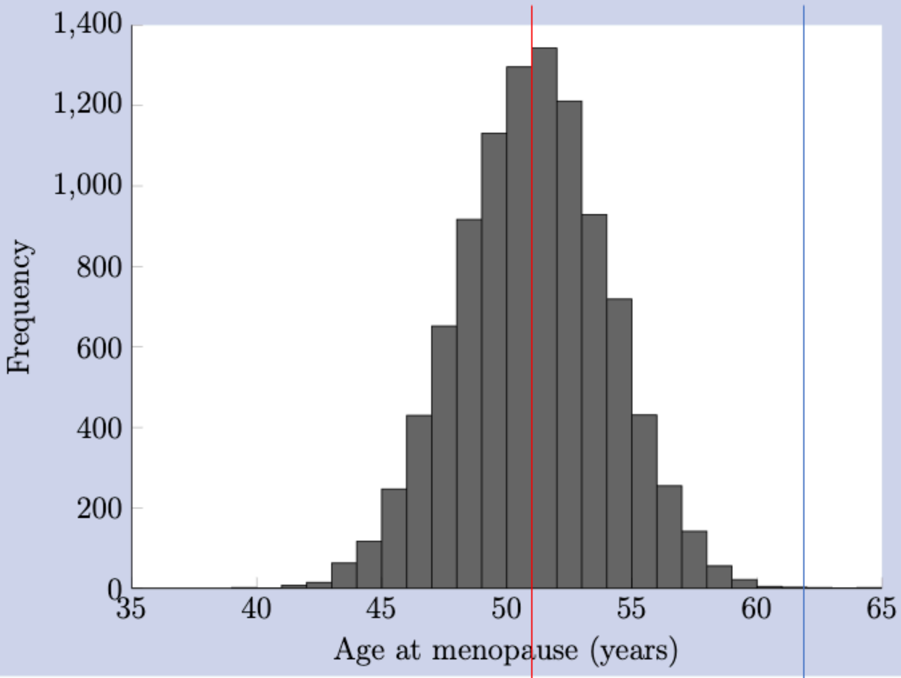
</td></tr>
<tr><td>Subject-Variable, Time-Invariant
(Figures 2b, 3a)</td><td>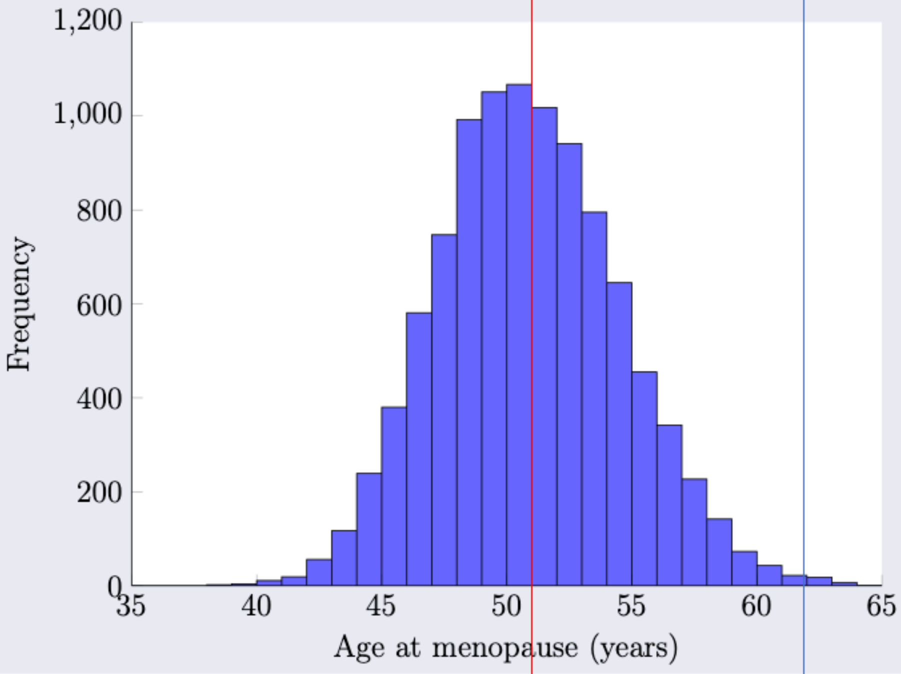
</td></tr>
<tr><td>Subject-Variable, Time-Variant*
(Figure 3c)</td><td>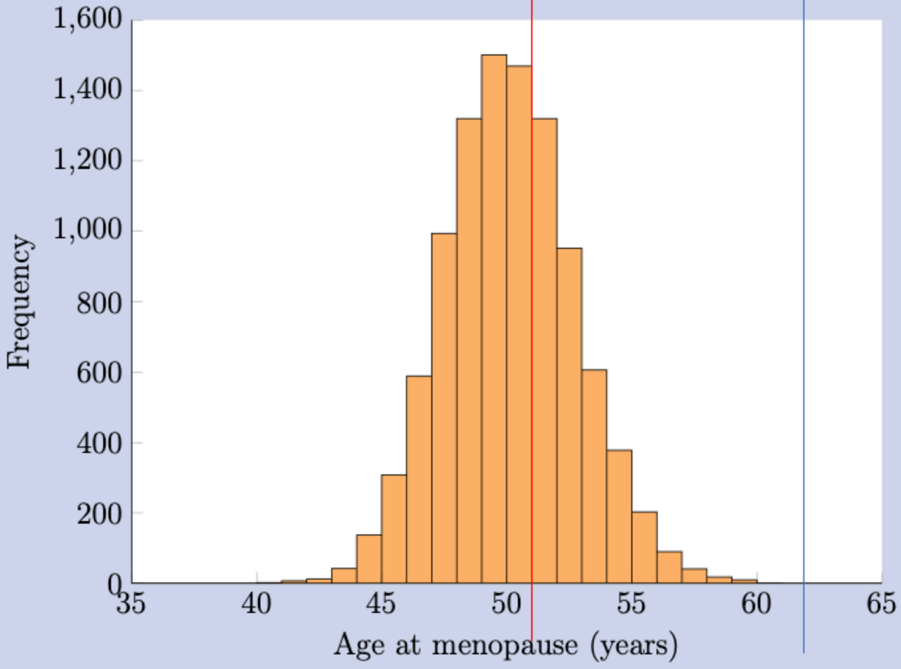
</td></tr>
</table>
