# Supplementary material for: Recapitulating human ovarian aging using random walks
Source: PeerJ. 2022 Aug 22;10:e13941. doi: 10.7717/peerj.13941 (PMC9406804; doi:10.7717/peerj.13941)
Supplement: Supplemental Information 3 — We first expand upon the alignment between continuous and discrete random walk models, then provide the justification for treatment of PF starting supply as a log-normal distribution (Fig. S1), and finally provide ANM simulation results as histograms for further consideration (Fig. S2). [file peerj-10-13941-s003.pdf]

APPENDIX

Supplemental information in support of our mathematical modeling approach is provided as follows. We first expand upon the alignment between continuous and discrete random walk models, then provide the justification for treatment of PF starting supply as a log-normal distribution (Fig. S1), and finally provide ANM simulation results as histograms for further consideration.

DISCRETE AND CONTINUOUS RANDOM WALK MODELS

We now show the equivalence of the discrete random walk in (1) and the continuous random walk in (2) for small steps Δt and Δx. The discrete random walk in (1) can be written as

$$X((n+1)\Delta t) = X(n\Delta t) + \Delta x\, \xi_{n+1}, \quad n \geq 0, \tag{12}$$

where $\{\xi_n\}_{n\geq 1}$ is an independent and identically distributed (iid) sequence of random variables with

$$\xi_n = \begin{cases} +1 & \text{with probability } p, \\ -1 & \text{with probability } 1-p. \end{cases}$$

Defining D and V as in (3), the discrete random walk (12) can be written as

$$X((n+1)\Delta t) = X(n\Delta t) - V\Delta t + \sqrt{2D\Delta t}\, Z_{n+1}, \quad n \geq 0, \tag{13}$$

where

$$Z_n := \xi_n - 2p + 1, \quad n \geq 1.$$

Notice that $\{Z_n\}_{n\geq 1}$ is an iid sequence with

$$\mathbb{E}[Z_n] = 0, \quad \text{Variance}(Z_n) = 4p(1-p).$$

If we take $\Delta x \to 0$, $\Delta t \to 0$, and $p \to 1/2$ while keeping D and V in (3) fixed, applying the functional central limit theorem (Billingsley, 1995) to (13) yields that the discrete random walk $\{X(n\Delta t)\}_{n\geq 0}$ converges in distribution to the continuous random walk $\{X(t)\}_{t\geq 0}$ process satisfying the stochastic differential equation in (2).

Reserve exit time τ

Exact probability distribution of τ

For the continuous random walk $\{X(t)\}_{t\geq 0}$ satisfying (2), the reserve exit time τ is the first time that the random walk leaves the interval $(0,L)$. Mathematically, this is denoted by

$$\tau := \inf\{t > 0 : X(t) \notin (0,L)\}. \tag{14}$$

Define the survival probability,

$$S(x,t) := \mathbb{P}(\tau > t \,|\, X(0) = x),$$

where we have conditioned on the initial position of the random walk. The survival probability $S(x,t)$ is the unique solution of the following backward Kolmogorov equation (Gardiner, 2009),

$$\frac{\partial}{\partial t} S = D \frac{\partial^2}{\partial x^2} S - V \frac{\partial}{\partial x} S, \quad x \in (0,L),\, t > 0, \tag{15}$$

with absorbing Dirichlet boundary conditions, $S(0,t) = S(L,t) = 0$, and unit initial condition $S(x,0) = 1$.

In order to solve for $S(x,t)$, we first define the solution operator for the partial differential equation in (23) subject to absorbing boundary conditions in the special case that $V = 0$ by $\Phi^t(q)$. That is, Φ^t is a linear operator that takes an initial condition, $q(x)$, and maps it to the solution of (23) with $V = 0$ subject to absorbing boundary conditions at time $t > 0$. It is straightforward to solve for Φ^t explicitly via a standard separation of variables calculation and find

$$(\Phi^t(q))(x) = \sum_{k=1}^{\infty} \langle \phi_k, q \rangle e^{-\nu_k t} \phi_k(x), \tag{16}$$

where the eigenvalues, $\{\nu_k\}_{k\geq 1}$, and orthonormal eigenfunctions, $\{\phi_k\}_{k\geq 1}$, are given by

$$\nu_k = \frac{Dk^2\pi^2}{L^2}, \quad \phi_k(x) = \sqrt{\frac{2}{L}}\sin\Big(\frac{k\pi x}{L}\Big), \tag{17}$$

and $\langle\cdot,\cdot\rangle$ denotes the inner product,

$$\langle f,g\rangle := \int_0^L f(x)g(x)\,dx.$$

It follows that

$$S(x,t) = e^{\frac{V}{2D}x}e^{-\frac{V^2}{4D}t}\Phi^t(e^{\frac{-V}{2D}z}). \tag{18}$$

To make (18) explicit, we first calculate the inner product

$$\begin{aligned}\langle\phi_k(x), e^{\frac{-V}{2D}x}\rangle &= \int_0^L e^{\frac{-V}{2D}x}\sqrt{\frac{2}{L}}\sin\Big(\frac{k\pi x}{L}\Big)\,dx\\ &= \frac{4\sqrt{2}\pi D^2 k\sqrt{L}\Big(1-(-1)^k e^{\frac{-LV}{2D}}\Big)}{4\pi^2D^2k^2+L^2V^2}, \quad k\geq 1.\end{aligned}$$

Therefore, (16) and (18) imply

$$S(x,t) = \sum_{k=1}^{\infty} A_k e^{-\lambda_k t}, \tag{19}$$

where

$$\begin{aligned}\lambda_k &:= \frac{Dk^2\pi^2}{L^2} + \frac{V^2}{4D}\\ A_k &:= \frac{4\sqrt{2}\pi D^2 k\sqrt{L}\Big(1-(-1)^k e^{\frac{-LV}{2D}}\Big)}{4\pi^2D^2k^2+L^2V^2}e^{\frac{V}{2D}x}\sqrt{\frac{2}{L}}\sin\Big(\frac{k\pi x}{L}\Big), \quad k\geq 1.\end{aligned}$$

Growth and death probabilities

In our model, a PF begins to grow if its ISR activity hits the growth threshold at $X = 0$ and it dies before beginning to grow if its ISR activity hits the death threshold at $X = L > 0$. For the parameter values in (6), the vast majority of PFs grow rather than die.

To study this quantitatively, define

$$\begin{aligned}\tau_0 &:= \{t > 0 : X(t) = 0\},\\ \tau_L &:= \{t > 0 : X(t) = L\}.\end{aligned}$$

In words, τ_0 is the first time the random walk reaches 0, and τ_L is the first time the random walk reaches L. Note that the reserve exit time τ in (14) is thus the minimum of τ_0 and τ_L. Hence, a PF dies before beginning to grow if $\tau_0 > \tau_L$ (i.e. if its ISR activity hits the death threshold at $X = L$ before the growth threshold at $X = 0$).

Define the probability that a PF dies before beginning to grow,

$$u(x) := \mathbb{P}(\tau_0 > \tau_L\,|\,X(0) = x), \tag{20}$$

where we have conditioned on the initial ISR activity, $X(0) = x$. The probability $u(x)$ satisfies Gardiner (2009)

$$0 = D\frac{d^2}{dx^2}u - V\frac{d}{dx}u, \quad x\in(0,L),$$

with boundary conditions $u(0) = 0$ and $u(L) = 1$. It is straightforward to check that the unique solution to this boundary value problem is

$$u(x) = \frac{e^{\frac{-V(L-x)}{D}} - e^{\frac{-VL}{D}}}{1-e^{\frac{-VL}{D}}}. \tag{21}$$

Evaluating (21) at the parameter values in (6) yields

$$u(x) = 2.05\times 10^{-6}. \tag{22}$$

Approximate probability distribution of τ

We have found that the vast majority of PFs hit the growth threshold before the death threshold. This suggests that we can approximate the probability distribution of τ by ignoring the death threshold. To study this case, define the survival probability,

$$S_0(x,t) := \mathbb{P}(\tau_0 > t \,|\, X(0) = x).$$

The survival probability $S_0(x,t)$ is the unique solution of the following backward Kolmogorov equation, (Gardiner, 2009)

$$\begin{aligned} \frac{\partial}{\partial t} S_0 &= D\frac{\partial^2}{\partial x^2} S_0 - V\frac{\partial}{\partial x} S_0, \quad x > 0, t > 0, \\ S_0 &= 0, \quad x = 0, \\ S_0 &= 1, \quad t = 0. \end{aligned} \tag{23}$$

A straightforward calculus exercise verifies that

$$S_0(x,t) = \frac{1}{2}\Big[1 + \text{erf}\Big(\frac{x - Vt}{\sqrt{4Dt}}\Big) - e^{Vx/D}\Big(1 - \text{erf}\Big(\frac{x+Vt}{\sqrt{4Dt}}\Big)\Big)\Big]$$

satisfies (23). Equation (7) then follows from (4) upon setting $x = 1$.

For the values of V and D in (6) with $x = 1$ and $L \geq 2$, the solution $S(x,t)$ is well-approximated by $S_0(x,t)$. Again, the basic reason is that for these parameter values, it is very unlikely for a PF to hit the death threshold at L before the growth threshold at 0. To make this precise, observe that

$$\begin{aligned} \mathbb{P}(\tau_0 > t) &= \mathbb{P}(\tau_0 > t, \tau_L > \tau_0) + \mathbb{P}(\tau_0 > t, \tau_0 > \tau_L) \\ &\leq \mathbb{P}(\tau_0 > t, \tau_L > t) + \mathbb{P}(\tau_0 > \tau_L) \\ &= \mathbb{P}(\tau > t) + \mathbb{P}(\tau_0 > \tau_L). \end{aligned}$$

Therefore,

$$0 \leq \mathbb{P}(\tau_0 > t) - \mathbb{P}(\tau > t) \leq \mathbb{P}(\tau_0 > \tau_L). \tag{24}$$

By definition of S and S_0, the bound (24) implies

$$0 \leq S_0(x,t) - S(x,t) \leq u(x), \tag{25}$$

where $u(x)$ is the probability in (20). Evaluating $u(x)$ at the parameter values in (6) as in (22), we obtain

$$0 \leq S_0(x,t) - S(x,t) \leq 2.05 \times 10^{-6}.$$

Starting supply distribution

We model the distribution of the starting supply N across a population of women as a log-normal distribution as in (8)-(9). The parameters μ and σ in (8) are the respective mean and standard deviation of the natural logarithm of the 30 PF counts in Wallace and Kelsey (2010) taken from women who were at least 6 months gestation and at most one month post birth. In Figure S1a, we plot the histogram of these 30 PF counts (blue bars), which is well-approximated by the probability density function of the log-normal distribution in (9) with μ and σ in (8) (dashed black curve). In Figure S1b, we plot the corresponding empirical cumulative distribution function for these 30 PF counts (solid blue curve) and the cumulative distribution function of the log-normal distribution in (8)-(9) (dashed black curve). The Kolmogorov-Smirnov distance between these two distributions in Figure S1b (i.e. the maximum absolute difference) is only 0.1, which has a corresponding p-value of 0.88 for the null hypothesis that these 30 PF counts are indeed sampled from the log-normal distribution in (8)-(9).

For starting supply, we chose to consider women who were within a few months of birth since only 15 PF counts in Wallace and Kelsey (2010) were from women at birth. However, considering only these 15 PF counts at birth would have little effect on our results, and would only change the values $\mu = 12.686$, $\sigma = 0.497$ in (8) to $\mu = 12.801$, $\sigma = 0.490$.

SUPPLEMENTAL FIGURES

Supplemental information in support of our mathematical modeling approach is provided as follows. First, we show how PF starting supply was determined according to the distribution of PF numbers around the time of birth produced by Wallace and Kelsey (2010) (Fig. S1).

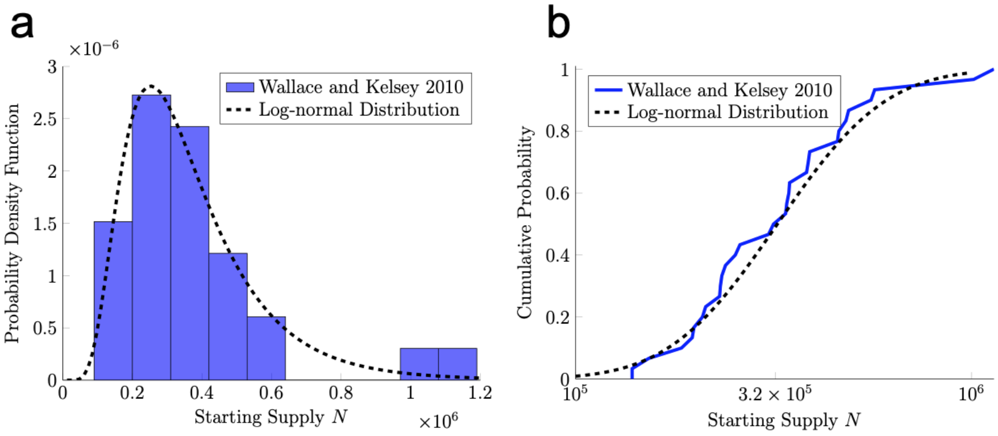

Appendix 1, Figure S1. Starting supply distribution. In panel a, we plot a histogram of the 30 PF counts for women near birth reported by Wallace and Kelsey (2010) (blue bars), which is well-approximated by the log-normal distribution in (8)-(9) (dashed black curve). In panel b, we provide a cumulative distribution function plot of observed PF counts (blue solid line) versus the log-normal distribution (dashed black curve).

ANM histograms in Fig. S2 correspond to data shown in Figures 1 and 2, with model conditions indicated by Figure panel.

Drift Parameters (Corresponding Figure)	ANM Histogram
Subject-Identical, Time-Invariant (Figure 2a)	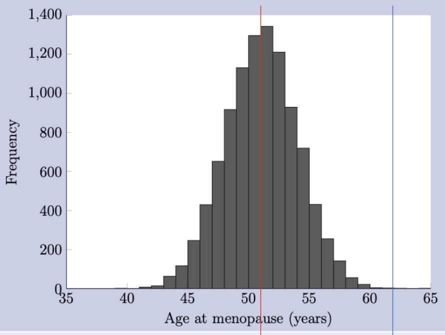
Subject-Variable, Time-Invariant (Figures 2b, 3a)	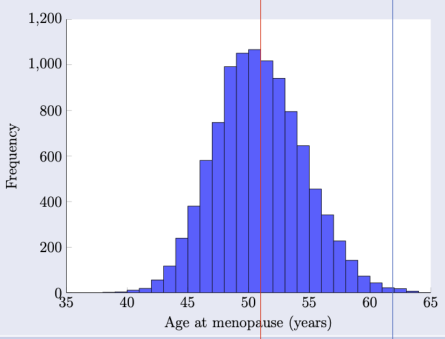
Subject-Variable, Time-Variant* (Figure 3c)	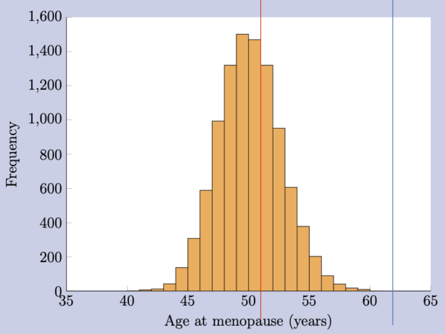

Appendix 1, Figure S2. ANM histograms generated from RW output when drift was set as specified in "Drift Parameters" column. As shown, an ANM distribution centered around a median age of approximately 51 (red vertical line) can be produced in each case, with few simulated subjects reaching menopause before 40 years and after 60 years. Time-Variant drift indicated by the asterisk (*) was applied by modifying drift conditions and also applying a single step drift acceleration in year 38 of simulation time. This was used to interrogate the possibility that PF loss accelerates during reproductive aging. Note that here, the ANM distribution generated when Subject-Variable drift is applied (middle panel) is broader than that seen for homogenous drift (top panel) given otherwise identical model conditions. Application of Time-Variant drift resulted again in a narrower ANM distribution, and prevented simulated subjects from reaching the ANM threshold after age 62 (blue vertical line).
